# Electrospun Nanofibers for Functional Food Packaging Application

**DOI:** 10.3390/ma16175937

**Published:** 2023-08-30

**Authors:** Meng Zhang, Adnan Ahmed, Lan Xu

**Affiliations:** 1 National Engineering Laboratory for Modern Silk, College of Textile and Engineering, Soochow University, 199 Ren-Ai Road, Suzhou 215123, China; zm1876395319@163.com (M.Z.); adnanahmed0070@outlook.com (A.A.); 2 Jiangsu Engineering Research Center of Textile Dyeing and Printing for Energy Conservation, Discharge Reduction and Cleaner Production (ERC), Soochow University, 199 Ren-Ai Road, Suzhou 215123, China

**Keywords:** electrospun nanofibers, food packaging, active packaging, indicators, antioxidant, antimicrobial

## Abstract

With the strengthening of the public awareness of food safety and environmental protection, functional food packaging materials have received widespread attention. Nanofibers are considered as promising packaging materials due to their unique one-dimensional structure (high aspect ratio, large specific surface area) and functional advantages. Electrospinning, as a commonly used simple and efficient method for preparing nanofibers, can obtain nanofibers with different structures such as aligned, core-shell, and porous structures by modifying the devices and adjusting the process parameters. The selection of raw materials and structural design of nanofibers can endow food packaging with different functions, including antimicrobial activity, antioxidation, ultraviolet protection, and response to pH. This paper aims to provide a comprehensive review of the application of electrospun nanofibers in functional food packaging. Advances in electrospinning technology and electrospun materials used for food packaging are introduced. Moreover, the progress and development prospects of electrospun nanofibers in functional food packaging are highlighted. Meanwhile, the application of functional packaging based on nanofibers in different foods is discussed in detail.

## 1. Introduction

Food is prone to decay during storage, and the environmental humidity, storage temperature, and external light conditions will affect the quality of food. Meanwhile, the growth of microorganisms, enzyme-catalyzed food decomposition, and oxidation of food in contact with air will also lead to the growth of mold, loss of moisture, poor food flavour, and so on [1]. Food packaging can create a barrier between the environment and food, thereby preventing food corruption caused by foreign substance contamination and protecting food from chemical, physical, and biological hazards [2]. Food packaging can be generally characterized as edible or non-edible films and coatings. Films can be obtained by solvent casting, extrusion, and electrospinning. Coatings are directly applied to food surfaces by dipping, spraying, painting, or panning. Edible packaging, such as sugar coatings on tablets, gelatin films used on drug capsules [3], edible coatings on cheeses and fruits [4], and bio-based foams and hydrogels [5], can avoid packaging waste and reduce environmental damage. Plastic bags, plastic film, plastic wraps, and other types of plastic products are non-edible packaging. Further processing of plastic sheets using thermomechanical methods, such as thermoforming, extrusion blow molding, and blow molding, can form more complex packaging.

Currently, with the increasing demand for food safety and environmental protection, the use of natural biological and biodegradable packaging materials has increased. Moreover, a series of functional food packaging is being developed to retard food deterioration, improve food sensory properties, and ensure food safety. The ideal functional food packaging has enhanced antibacterial, antioxidant, water resistance, and other properties by adding some active substances or using physical and chemical modification (such as heat treatment, plasma treatment, and radiation treatment) [6], thus maintaining the quality and sensory properties of the food, improving food safety, and effectively prolonging the shelf life of food. In addition, food packaging can also act as an intelligent monitoring system to monitor the quality changes in food during storage in real time, thereby reminding consumers of the real-time quality of food and bringing about more healthy consumption for people [7]. 

In order to meet the changing needs of food packing, it is important to adopt nanotechnology and find new food packaging technologies [8,9]. Compared with bulk materials, nanomaterials have better physical, chemical, optical, mechanical, and catalytic properties, which makes the application of nanotechnology in food packaging very promising [10]. A recent development is the integration of active agents into packaging materials to improve food safety and quality. In particular, the combination of antioxidants and packaging materials will resist the deterioration of physical properties such as the flavor and color of food [11,12]. It is well known that polymers are the preferred materials for active food packaging because of their functional properties as well as their ability to carry active agents and their controlled release [13]. However, most active agents evaporate easily due to their high volatility [14], making it impossible to directly inject them through typical processing methods of polymers [15]. To overcome this problem, electrospinning (ES) technology can be applied to food packaging [16]. 

ES technology has been widely used in the manufacture of nanofibers due to its simple operation, low cost, wide application scope, and high production efficiency [17,18]. This technology can not only control the orientation, shape, and structure of nanofibers by modifying devices and adjusting process parameters, but also load various functional substances into nanofibers [19]. Furthermore, the ES process does not involve high temperature conditions, which can ensure the stability of active substances. Moreover, the solvent in the spinning solution will evaporate quickly during the ES process, greatly reducing the safety problems caused by toxic solvents in electrospun nanofibers [20]. Meanwhile, electrospun raw materials for food packaging are widely available [21]. These advantages of ES technology provide a good basis for the application of electrospun nanofibers in food packaging, and have attracted a great deal of attention [22]. Electrospun nanofibers have a high aspect ratio, large specific surface area, good physical and chemical properties, and will not be deformed under high temperature conditions. Electrospun nanofiber membranes (NFMs) used in food packaging are generally composed of polymers with good flexibility, fine feel, good air permeability, easy degradation, and low wear. NFMs have a large specific surface area and high porosity, which are suitable for the release of active substances [23]. Moreover, electrospun NFMs have good barrier properties, which can improve the closure of food packaging, reduce its permeability, and effectively inhibit the growth of microorganisms. They can also independently choose to filter oxygen and carbon dioxide, forming a natural air-conditioned packaging for fruits and vegetables to prolong the freshness of food. Therefore, electrospun NFMs have shown great potential for application in the field of food packaging.

## 2. Electrospinning Technology

**Figure 1:**
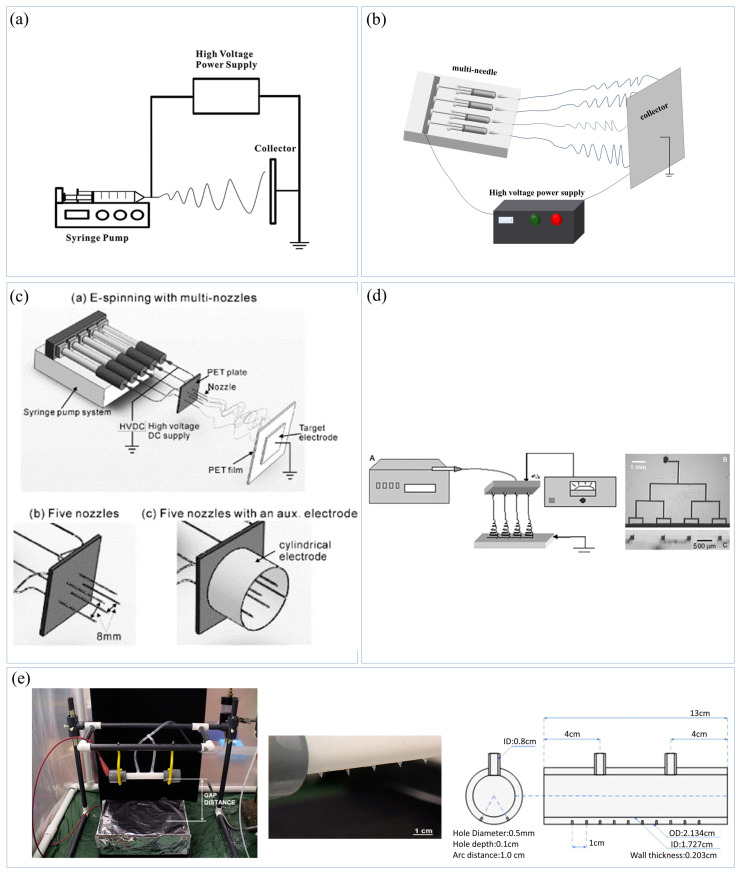
Schematic diagrams of needle electrospinning devices. (**a**) Traditional single-needle electrospinning device [27], (**b**) multi-needle electrospinning device, (**c**) auxiliary electrodes or additional electrodes are used in the multi-needle electrospinning [24], (**d**) multi-nozzle microfluidic electrospinning device ((**A**) Schematic diagram of microfluidic electrospinning using a branching microchannel architecture to simultaneously spin multiple fibers directly from the channel outlets. (**B**) Close-up of top side of the poly(dimethy-siloxane) (PDMS) device with channels filled with food coloring to enhance contrast. (**C**) Side-on cross-section of the device, showing the channel outlets.) [28]. (**e**) A multi-needle electrospinning device with 20 holes in the hollow tube as a spinneret [29].

ES technology refers to the process in which polymer solutions or melts overcome their surface tension under a high-voltage electric field, achieve jet stretching, and solidify into nanofibers after solvent evaporation or melt cooling [24,25,26]. The traditional single-needle ES is mainly composed of three parts, namely, reservoir area, spinning area, and collector (Figure 1a) [27]. Despite its simple structure and low operational difficulty, its spinning efficiency is low (0.01–0.1 g/h), limiting its industrial application [19]. The multi-needle ES realizes mass production of nanofibers by increasing the number of needles (Figure 1b) [25]. These needles are generally arranged in certain shapes, such as linear, circular, oval, etc. [26]. However, the interaction between the needles and the non-uniformity of the electric field at the needle tips are the two most serious problems in the multi-needle ES. To solve the problems, auxiliary electrodes or additional electrodes are used in the multi-needle ES. Kim et al. [24] placed a cylindrical auxiliary electrode outside the five needles to shorten the chaotic motion of the jets and produce thicker fibers (Figure 1c). In addition, the holes and channels of spinnerets can be applied as needles, and a multi-needle ES is called a porous ES. Srivastava et al. [28] established a multi-nozzle microfluidic ES device, which used micro-channels to transport the solution (Figure 1d). Varabhas et al. [29] reported a hollow tube with 20 holes (i.e., nozzles) as a spinneret (Figure 1e). Compared with a multi-needle ES, a porous ES takes up less space and is easy to operate. However, in porous ES, the arrangement and spacing of the nozzles are not easily changed because they are usually fixed on a spinneret, and jet interference and nozzle clogging are still difficult problems [30]. 

**Figure 2:**
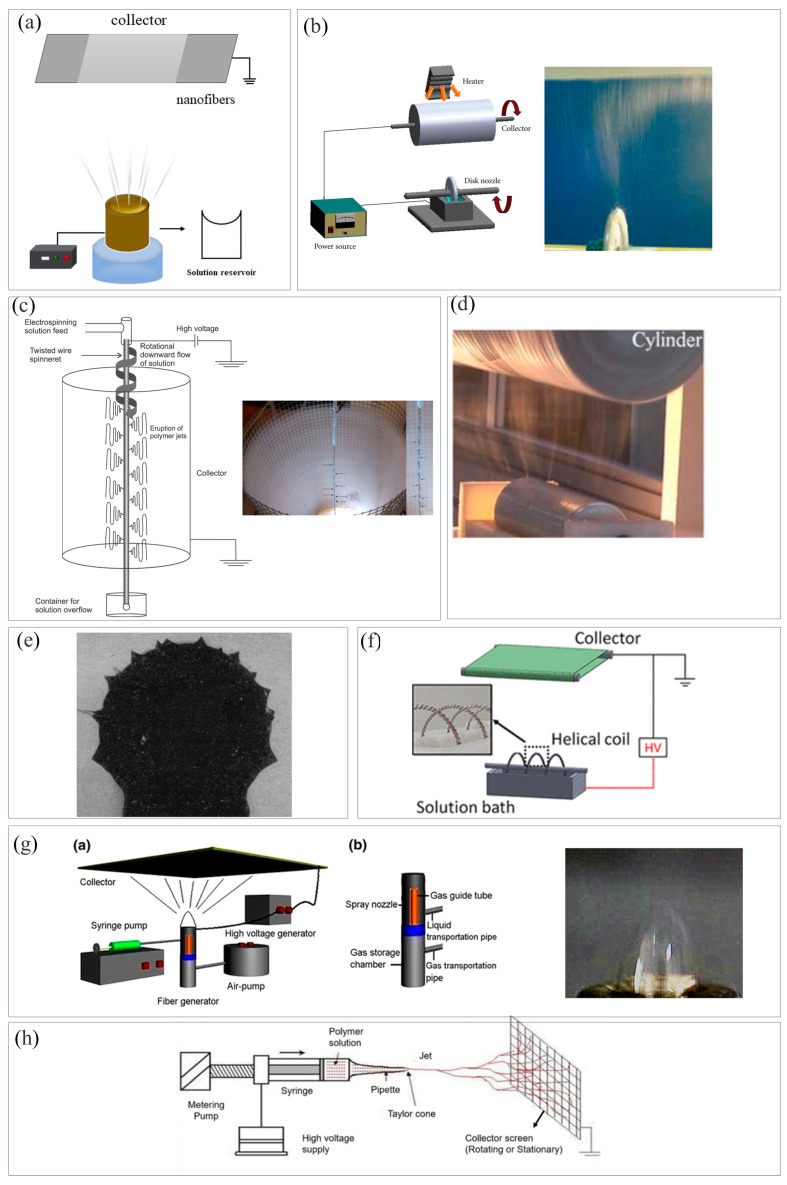
Schematic diagrams of needle-free electrospinning devices. (**a**) Needle-free electrospinning, (**b**) disc spinneret [31], (**c**) wire spinneret [32], (**d**) cylinder spinneret [33], (**e**) ball spinneret [34], (**f**) coil spinneret [35], (**g**) bubble electrospinning (Schematic image of the modified air-jet electrospinning setup (**a**) and structure parts of fiber generator (**b**)) [36], (**h**) electrospinning device with a changed spinneret [37].

Accordingly, needle-free ES provides another method to enhance the yield of nanofibers, in which multiple jets are generated directly from the open solution surface without being affected by the electric field interference between multiple needles (Figure 2a). According to the different spinnerets, needle-free ES devices can be divided into two types: rotating and stationary. The spinning process of rotating needle-free ES is mainly achieved by rotating the spinnerets (such as disc (Figure 2b) [31], wire (Figure 2c) [32], cylinder (Figure 2d) [33], ball (Figure 2e) [34], or coil (Figure 2f) [35]) in the reservoir, forming many small droplets, similar to Taylor cones, on the solution surface. The spinning process of stationary needle-free ES is mainly achieved through the direct or other auxiliary methods (such as air flow (Figure 2g) [36] or changing spinneret structure (Figure 2h) [37]) to overcome the solution surface tension, thus forming many small protrusions, similar to Taylor cones, on the solution surface. Then, these small droplets or protrusions generate multiple jets under the action of electric field force. Finally, these jets are stretched and solidified to form a large number of nanofibers. In addition, Demir et al. [38] found the number of jets increased with enhancing voltage. Kong et al. [39] observed that under a high voltage and low solution concentration, the solution surface tended to form convex curvature droplets, thereby generating multiple jets. The yields of various ES devices are shown in Table 1.

## 3. Electrospun Raw Materials for Food Packaging

**Figure 3:**
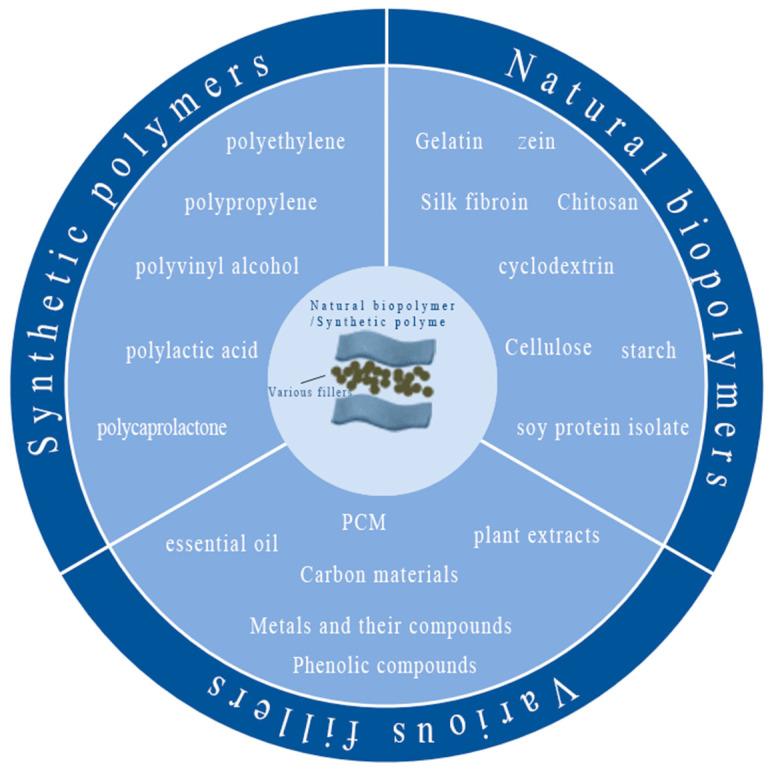
Electrospun raw materials used for food packaging.

The raw materials of electrospun NFMs commonly used for food packaging are polymers, mainly including synthetic polymers and natural polymers [23,51,52,53]. Synthetic polymers are the main base materials for food packaging. However, some synthetic polymers are limited in resources and do not easily degrade [54]. For environmental protection, many reliable, safe, and biodegradable raw materials in the natural environment can be used for food packaging production through ES technology [55,56,57], and various fillers can be loaded into the base materials to enhance the mechanical, antimicrobial, and antioxidant properties of food packaging [58], as shown in Figure 3 and Table 2.

### 3.1. Natural Polymers

Natural polymers, such as protein and sugar, are generally used as base materials in food packaging [55,56,57]. However, their low mechanical strength and poor spinnability limit their application in food packaging [89]. Food packaging materials generally require good hydrophobicity, with a WCA value greater than 90° [90], while most natural polymers have poor hydrophobicity. Therefore, their application in food packaging can be expanded by adding various fillers or combining them with other spinnable polymers. 

#### 3.1.1. Protein

Most proteins have poor spinnability. Poor spinnability of solutions means that spinning solutions cannot easily spin nanoscale fibers with good morphology, which may be due to the low conductivity or high viscosity of the spinning solutions. Moreover, most proteins have poor mechanical properties and hydrophobicity, which means that they do not perform well when used alone for packaging. Zein is a water-insoluble alcohol soluble protein from maize, which has good barrier properties for transport of gases, water vapor, or solutes, as well as good biocompatibility and biodegradability. However, when the zein concentration in the spinning solution is less than or equal to 10%, it is impossible to electrospin nanofibers. In recent years, zein films have already been applied as edible coatings on nuts to delay rancidity, and on tomatoes to delay color changes and weight loss and to maintain firmness during storage [91]. Zein-based NFMs prepared by ES have been widely used in food package. Roberta et al. [92] electrospun vanillin/zein NFMs, where the vanilla powder encapsulation rate reached 74%, as an alternative to synthetic polymers used in commercial applications of food packaging.

Gelatin is a safe and harmless protein extracted from collagen, which has excellent barrier, mechanical, biodegradable, and biocompatible properties. It is widely used in the food, medicine, and cosmetics industries. However, gelatin has strong hydrophilicity, with a WAC of about 45°, and cannot protect food from water vapor for a long time [93]. Accordingly, other hydrophobic polymers or active substances are commonly used to mix with it for spinning so that the NFMs prepared can further extend the shelf life of food [68]. Tang et al. [94] used ES technology to prepare gelatin NFMs containing peppermint essential oil or chamomile essential oil. It was found that the addition of essential oil increased the hydrophobicity of the NFMs, improved the antibacterial activity of the NFMs, and compensated for the limitations of gelatin’s properties. Gelatin nanofibers containing 9% *v/v* chamomile essential oil turned out to be hydrophobic with a WAC of 101.3 ± 4.3°.

Soybean protein (SPI) has good biocompatibility, low cost, good and smooth film-forming properties, transparency and flexibility, but it is a globular protein with low solubility in organic solvents and poor mechanical properties, which complicates fiber formation through electrospinning. Daehwan et al. [95] electrospun SPI/PVA NFMs, and it was found that the mechanical properties of pure PVA NFMs were the best, and the mechanical properties of electrospun nanofibers decreased with the increase in SPI content. SPI is proposed as an excellent carrier material. Bruni et al. [96] formulated a hybrid emulsion of SPI and PVA, added the antioxidant β-carotene to the emulsion, and then prepared a packaging coating using ES. The experiments proved that an SPI emulsion could effectively encapsulate antioxidants, and could persistently release bioactive compounds when packaging food with it.

SF is a natural polymer fibrin extracted from silk, which has good physical and chemical properties as well as biocompatibility. Especially, SF has the functions of oxidation resistance and acting as a water vapor barrier. When it is used in packaging materials, it can effectively improve the shelf life of food [97]. However, due to its poor spinnability and strong hydrophilicity, it is often mixed with other materials to improve the NFM’s performance. Lin et al. [73] prepared SF nanofibers containing thyme essential oil. When PEO was added to the SF solution at a ratio of more than 20%, stable nanofibers could be prepared by ES. After plasma treatment, the NFMs could effectively release thyme essential oil to kill *Salmonella typhi*, which was an effective antibacterial packaging to extend the shelf life of food.

#### 3.1.2. Sugar

Starch is a kind of natural polysaccharide, which has the characteristics of low cost, easy processing, being renewable, etc. Starch-derived edible food films have great potential as biodegradable food packaging materials because they reduce the overuse of traditional petroleum-based plastic [98]. However, there are a large number of hydrophilic hydroxyl groups in the starch molecule; the performance of starch NFMs for food packaging application can be improved by using self-assembly, grafting, and cross-linking. In order to overcome the problem of the ultra-low hydrophobicity of electrospun starch NFMs, Cai et al. [98] prepared a stearic acid (STA) coating through solution immersion, and changed the hydrophobicity of the NFMs by controlling the assembly of this coating on the surface of starch NFMs. The WAC of the NFMs increased from approximately 0° to 134.7° before and after coating the starch NFMs with STA. Zhang et al. [71] prepared starch/tea polyphenol NFMs and experimentally demonstrated that the addition of tea polyphenols endowed the NFMs with antioxidant properties, and with the increase in the cross-linking time from 0.5 to 2.5 h between tea polyphenols and starch, the WAC of the NFMs was significantly improved from 17.5° to 87.2°, and there was no negative impact on the antioxidant properties of the NFMs. 

Cellulose is the most widely distributed and abundant polysaccharide in nature, being the main component of plant cell walls [99]. There are many kinds of cellulose, including carboxymethyl cellulose (CMC), hydroxypropyl cellulose (HPMC), ethyl cellulose, and cellulose acetate (CA). CMC has good biocompatibility and biodegradability, but it is easily soluble in water and has poor spinnability. Hashmi et al. [75] prepared PVA/polyvinylpyrrolidone (PVP)/CMC nanofibers by ES, and found that these nanofibers had a homogeneous morphology and exhibited better mechanical properties (tensile strength over 10 MPa) and hydrophobicity due to the cross-linking between PVA, PVP, and CMC. The WACs of PVA and PVA/PVP were 87.7° and 80.4°, respectively, and the WAC of PVA/PVP/CMC NFM increased to about 100° with the addition of CMC. Therefore, these nanofibers were used in food packaging to keep food fresh. HPMC has good film-forming ability, biocompatibility, and degradation ability. Aydogdu et al. [54] directly deposited HPMC nanofibers on polylactic acid (PLA) NFMs to generate double-layer NFM-based packaging, which was a preparation method to reduce the transparency of NFM-based packaging without increasing its permeability. Ethyl cellulose is a kind of artificially modified cellulose with a low manufacturing cost, excellent mechanical properties, and strong water resistance. It can be blended with other hydrophilic polymers to improve the mechanical properties and water resistance of NFMs. Niu et al. [100] prepared zein/ethyl cellulose/cinnamon essential oil nanofibers. The WAC of zein NFM was 54° at 1 s and 16° at 60 s. The WAC of ethyl cellulose NFM was 131°, which barely changed with time. After adding ethyl cellulose to zein NFMs, hydrogen bonds were formed between hydroxyl groups of ethyl cellulose and amino groups of zein, reducing the number of free hydrophilic groups and improving the water resistance of the NFMs. CA is formed by acetylation of cellulose, which is a low-cost cellulose derivative with good mechanical properties, biodegradability, and biocompatibility. Tarus et al. [77] prepared poly (vinyl chloride) (PVC)/CA/Ag NFMs, which had good tensile properties and an excellent antibacterial effect. 

Chitosan (CS) is a safe, cheap, non-toxic natural polymer with excellent antibacterial effect, biocompatibility, and biodegradability. However, it is a cationic polymer with high density charges, which leads to high repulsion between its ionic groups, low spinnability, and poor mechanical properties produced when ES CS solutions [101]. In order to improve the spinnability of CS and the mechanical properties of NFMs, CS can be blended with other natural or synthetic polymers to prepare NFMs. Deng et al. [102] prepared CS/PEO/laurate arginine NFMs using ES, which exhibited an ultra-fine 3D porous structure and had excellent antibacterial activity. Duraiarasan et al. [103] prepared CS/PEO NFMs to encapsulate pomegranate peel extract. With the increase in CS content, the viscosity of the solution gradually increased and the fiber presented a bead shape. It was found that the composite fiber showed excellent mechanical properties and thermal stability. The tensile strength of pure PEO NFM was 2.4 ± 0.56 MPa. With the increase in the CS proportion, the tensile strength of CS/PEO NFM also increased, reaching a maximum of 10.8 ± 2.2 MPa. Furthermore, the NFM had an antibacterial effect on *Escherichia coli* (*E. coli*). The bacterial growth on beef was slower at 4 °C than at 25 °C. During psychrophilic storage, the total bacterial count reached 6.60 log cfu/g on the control, whereas for CS/PEO NFMs it reached only 2.96 log cfu/g after 10 days of storage, which showed it had great application potential in food packaging. 

**Figure 4:**
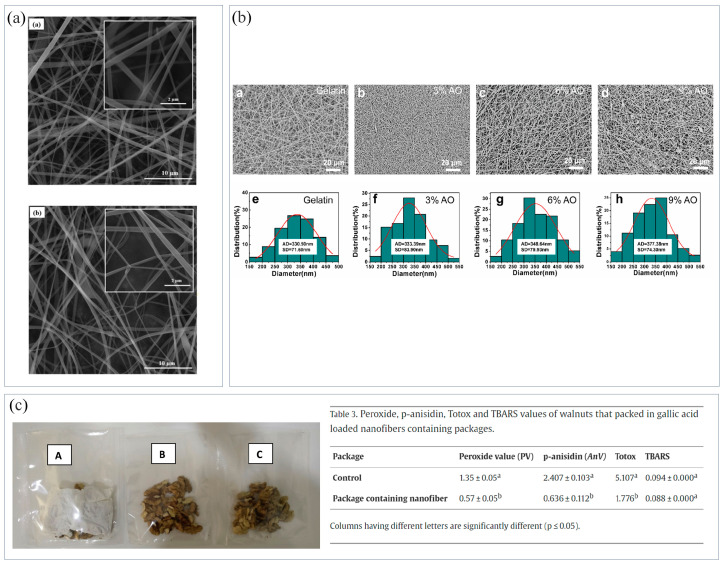
(**a**) SEM images and average fiber diameter of fibers: (**a**) hydroxypropyl-β-cyclodextrin (β-CD) fibers; and (**b**) cuminaldehyde/β-CD inclusion complex (CUM/β-CD) fibers [79]. (**b**) The addition of angelica essential oil (AO) increased the diameter of gelatin nanofibers [68]; (**c**) NFMs loaded with gallic acid for packaging walnuts: (left figure; **A**) 10% galllic acid loaded nanofiber, (**B**,**C**) control [81].

Cyclodextrin is a cyclic oligosaccharide which is often used to encapsulate various drugs in food packaging research. Wen et al. [78] encapsulated cinnamon essential oil (CEO) into a β-cyclodextrin (β-CD) inclusion complex to prepare biodegradable antibacterial materials and improve the antibacterial activity of nanofibers. Sharif et al. [79] prepared β-CD NFMs containing cuminaldehyde. The experiment proved that acrolein was encapsulated in β-CD at a very high encapsulation rate, and the encapsulated nanofibers had a uniform morphology (Figure 4a). After high temperature treatment, the weight loss rate of cuminaldehyde/β-CD NFMs was smaller than that of cuminaldehyde and β-CD, indicating an improvement in the thermal stability of the NFMs. Culturing *E. coli* and *Staphylococcus aureus* (*S. aureus*) on cuminaldehyde/β-CD NFMs, the results showed that the surviving populations of both microorganisms were obviously reduced for cuminaldehyde/β-CD fibers, indicating the inhibition of bacterial growth.

### 3.2. Synthetic Polymers

Many bio-based polymers have poor spinnability, and the morphology and properties of electrospun nanofibers produced using them are poor, limiting their application in food packaging. Synthetic polymers have the advantages of a low cost, easy production, being lightweight, and good flexibility, and their moderate addition can improve the mechanical properties of packaging materials, which are widely used in the production of disposable packaging.

#### 3.2.1. Non-Degradable Polymers

Conventional packaging materials, including petroleum-based plastics, paper, metal, and glass, are used in food packaging. Most petroleum-based plastics belong to non-degradable polymers and have the characteristics of rigidity, flexibility, barrier properties, low cost, and ease in processing, making them one of the most extensively used packaging materials. Common petroleum-based plastics include polyethylene, polypropylene, PVC, etc. [104]. PVC is generally made using the thermoplastic method with the advantages of easy processing, being non-flammable, non-deformable, and low cost, and is often applied as cling films in the agricultural and processed food markets. Tarus et al. [77] used ES technology to prepare PVC/CA/Ag NFMs with enhanced tensile properties and antibacterial effects. The tensile characteristics of flexible packaging materials can be described as inferior (<1 MPa), marginal (1–10 MPa), good (10–100 MPa), or superior (100 MPa). Accordingly, the tensile characteristics of CA electrospun NFMs with an average strength of 8.6 MPa could be described as marginal, while PVC NFMs had an average strength of 13 MPa, exhibiting good mechanical properties as a packaging material. PVC/CA NFMs loaded with Ag nanoparticles (NPs) displayed inhibited growth of yeast and mold after the incubation period. After growing bacteria on the NFMs, the number of fungi on PVC/CA NFM was about 0.4 cfu/cm^2^, while that on Ag NPs loaded NFM was only about 0.1 cfu/cm^2^.

#### 3.2.2. Degradable Polymers

Although adding non-degradable synthetic polymers can improve the spinnability of spinning solutions, they are terrible for environmental protection. Therefore, degradable synthetic polymers are more widely used in electrospun nanofiber-based food packaging, commonly including polycaprolactone (PCL), PLA, and PVA, etc. 

PCL is a fossil derived polymer with excellent biocompatibility, biodegradability, and non-toxicity, which can be combined with other types of polymers to enhance its application performance. Beikzadeh et al. [105] electrospun PCL/ethyl cellulose/gelatin/zataria multiflora essential oil (ZEO)/ZnO NP NFMs, which had appropriate biocompatibility on account of a cell viability obtained above 80% at designated times. The NFMs had high cell survival, and can be used in food packaging. Liu et al. [106] used hydrophobic PCL to encapsulate hydrophilic anthocyanin, preparing hydrophobic food packaging for monitoring the freshness of food. 

PLA is one of the most studied degradable polymers used to develop antimicrobial materials in recent years; it can extend the shelf life of food. In food packaging, various active drugs are often encapsulated into PLA fibers to obtain antimicrobial packaging materials. Vidal et al. [74] electrospun core-shell NFMs based on lauryl arginine ethyl ester (LAE), cellulose nanocrystals (CNCs), and PLA. Here, PLA effectively coated antibacterial drugs, slowed down the rate of drug release, and enhanced the antibacterial properties of packaging materials. 

PVA has excellent mechanical properties, good biodegradability, and biocompatibility, and can effectively block gas. Narayanan et al. [107] prepared PVA NFMs coated with γ-cyclodextrin and ferulic acid (FA) which achieved effective release of FA while enhancing the thermal stability. PVA also has good hydrophilicity and can be highly soluble in water. It is often combined with other additives in food packaging and undergoes simple graft modification to improve the water resistance of NFMs. Yu et al. [84] electrospun PVA/clove oil (CO) NFMs and, through heat treatment, the hydroxyl group of PVA interacted with the carboxyl group of CA, thus effectively improving the thermal stability, mechanical properties, and water resistance of the NFMs.

### 3.3. Various Fillers

A variety of fillers have been added to NFMs for functional food packaging, improving qualities such as antibacterial, antioxidant, and vinyl degradation.

#### 3.3.1. Metals and Their Oxides

Metals or metal oxide NPs have good mechanical, thermal, antibacterial, barrier, and optical properties and have been used in food packaging in recent years. The interaction of electrons generated by these NPs with water or atmospheric oxygen produces reactive oxygen species (ROS) (hydroxyl radicals, superoxide anions, hydrogen peroxide) which can interact with bacteria to enhance the antibacterial activity of NFMs. Valerini et al. [86] sputtered and deposited Ag NPs on PCL NFMs, coating PCL with Ag NPs. This coating not only gave the fibers the antibacterial properties of Ag NPs, but also maintained the hydrophobicity of PCL. Kowsalya et al. [82] obtained stable Ag NPs using green synthesis from grape peel and incorporated them into PVA to prepare green organic nanofibers with good antibacterial properties. Zhang et al. [108] prepared NFMs with different ratios of TiO_2_ NPs using ES. NFMs with 5% TiO_2_ were measured to have high photocatalytic activity for the degradation of ethylene. Mayorga et al. [109] used compression molded poly (3-hydroxybutyrate-co-3-hydroxyvalerate) (PHBV) NFMs as a bottom layer, and coated them with PHBV-based NFMs containing CuO NPs to prepare bilayer structured NFMs, thus increasing their antibacterial activity. Rapa et al. [87] prepared NFMs containing PHBV and 1 wt% Fe-doped ZnO NPs, which had shown significant antibacterial effects against *pseudomonas aeruginosa*. 

However, the increased amount of ROS produced by metal oxide NPs can induce cell damage or oxidative stress, which is contrary to their positive use as antimicrobials and antioxidants. For example, kidney diseases and hepatic injury may occur as a consequence of a single oral dose of ZnO NPs. The gastrointestinal tract offers a chance for ZnO NP ingestion, which may readily pass through biological barriers and enter the circulatory system [110]. The use and discharge of titanium oxide can have an impact on people and nature, raising the risk of harm to the environment and human health [111].

#### 3.3.2. Carbon Materials

Carbon nanotube (CNT) is a good material for enzyme immobilization. Liu et al. [80] electrospun regenerated cellulose/carboxylated CNTs/graphene oxide (GO) NFMs to immobilize lysozyme. Carboxyl groups on CNTs (or GO) were activated to react with the nucleophilic amino functionality at the side chains of amino acids in lysozyme, realizing the immobilization of lysozyme and providing antibacterial properties for the composite NFMs. However, the prolonged exposure to carbon NPs outside NFMs may bind to proteins or other biomolecules in the body, enter the skin and bloodstream, and be toxic to the skin and lungs [112]. Graphene has outstanding mechanical flexibility, excellent electrical and thermal conductivity, as well as optical transparency [113,114]. It has multiple conservation functional groups, which can interact with polar solutions or polymers to promote the dispersion of graphene in the polymer matrix [115]. Sergio et al. [116] embedded graphene into poly (ethylene-co-vinyl alcohol) and found that graphene could be effectively dispersed in the polymer. The composite NFM had high conductivity and could be used as a smart label in food packaging. 

#### 3.3.3. Phase Change Materials (PCMs)

PCMs are substances that undergo phase change at a specific temperature. When the external temperature changes, they can absorb and release heat energy, thus maintaining a constant temperature of food during food transport and storage [117]. However, due to their poor thermal stability and low thermal conductivity, PCMs cannot be directly used in packaging [100]. Encapsulating PCMs in a polymer through ES technology can effectively protect them from external influences [118]. Wilson et al. [119] used polystyrene (PS) as an encapsulation base for the commercial PCM RT15. RT15 could be effectively encapsulated in the PS matrix with an encapsulation rate of 78%, thereby improving its heat storage performance and maintaining the quality of packaged refrigerated food. Rocio et al. [120] encapsulated dodecane in PCL/PLA with a submicron droplet size using the ES technique, thus improving the thermal storage capacity of dodecane and maintaining food quality when packaging temperature-sensitive products with the electrospun NFMs. 

#### 3.3.4. Bioactive Compounds

Aromatic herbs are effective antimicrobial agents and antioxidants, containing beneficial phytochemicals such as terpenoids, phenols and derivatives, flavonoids, coumarins, quinine, saponins, tannins, and alkaloids, etc. [121]. These compounds are derived from plants and are generally less toxic to mammals and humans [122,123]. Accordingly, plenty of studies have focused on the extraction of bioactive compounds from plants to be applied in food packaging [124]. 

Among natural bioactive drugs, essential oils have the advantages of good biodegradability, antibacterial and antioxidant properties, and few side effects, and so are widely used in food packaging. The hydrophobic characteristics of essential oils enable them to partition into the cell membranes and mitochondria of the lipid layer, altering cell permeability and disrupting the cell wall structures, thus causing crucial molecules and ions to leak from the bacterial cells, thus leading to bacterial death [125]. However, the volatility, strong smell, and other problems of essential oils also limit their application in food packaging. Their long-term release can be effectively controlled by immobilizing them on a polymer matrix through ES [123]. Liu et al. [124] used the emulsion ES method to encapsulate cinnamon essential oil (CEO) into CS nanofibers. With time, CEO diffused to the fiber surface, and its release gradually increased. CS and CEO had a synergistic antibacterial effect, maintaining antibacterial activity as CEO slowly released. Fonseca et al. [72] encapsulated thyme essential oil in starch nanofibers, with an encapsulation rate of about 99%, and it effectively expressed antioxidant activity. Zhou et al. [68] prepared gelatin nanofibers containing angelica essential oil (AO). The addition of AO increased the diameter of gelatin nanofibers (Figure 4b), improved their hydrophobicity, and enabled the nanofibers to have good antioxidant activity and inhibit Gram-negative and -positive bacteria. 

Phenolic compounds are widely found in fruits, vegetables, cereals, and tea, and have a variety of biological activities, such as anticancer, antitumor, antioxidation, etc. Their common feature is that all molecules contain phenol groups, thus possessing antioxidation properties [126]. Dumitriu et al. [85] prepared PCL/vitamin E (α-tocopherol) NFMs using ES, which had good antioxidant properties. Phenolic compounds not only have antioxidant properties, but also have antibacterial and antifungal properties. Phenolic compounds in microalgae can effectively replace synthetic compounds. Kuntzler et al. [66] prepared CS/PEO NFMs coated with microalgae phenols, which had a good inhibitory effect on *S. aureus*. Gallic acid has good bioactivity, antibacterial, and antioxidant properties, however, it is sensitive to temperature, pH, oxygen, and light, and tastes bitter, which limits its use in food packaging. Aydogdu et al. [81] encapsulated gallic acid in HPMC/PEO NFMs using ES. The bioactivity of gallic acid was effectively retained in the NFMs, which could extend the shelf life of food. The NFM loaded with 10% gallic acid were chosen to package walnuts due to its higher loading efficiency and antioxidant activity (Figure 4c). Curcumin is a natural yellow-orange polyphenol compound with low molecular weight, which has good anti-inflammatory and antiviral effects. In addition, it is also an effective antibacterial agent and antioxidant. Wang et al. [61] prepared curcumin/zein NFMs, which exhibited excellent antioxidant and antibacterial activities against *E. coli* and *S. aureus*. Curcumin is also a natural food colorant which can be used to detect food deterioration. Luo et al. [127] electrospun curcumin/zein NFMs to effectively monitor the freshness of food. Tea polyphenol is a kind of polyphenol compound extracted from tea which is a non-toxic antioxidant and can effectively delay the deterioration of food. Zhang et al. [71] prepared starch/tea polyphenol NFMs using ES. Cross-linking between tea polyphenol and starch improved the hydrophobicity of NFMs, while the addition of tea polyphenol gave the NFMs antioxidant activity. Jaboticaba is a natural fruit rich in anthocyanins which has a high antioxidant and antibacterial effect. Avila et al. [128] extracted bioactive compounds from Jaboticaba peels using an immersion method. The extracts contained high amounts of phenolic substances and anthocyanins, and the prepared NFMs containing the extracts had excellent antibacterial and antioxidant properties. Aloe vera is an ancient medicinal plant which is widely used in the medical and cosmetic industries. Solaberrieta et al. [88] obtained NFMs with antioxidant activity by adding aloe vera extract to PEO. Bitter orange is a citrus plant containing phenols, flavonoids, and vitamins, which has excellent antioxidant properties. Rashidi et al. [70] prepared ethyl cellulose/SPI/bitter-orange-peel-extract NFMs. The NFMs with 20% concentration of the extract had high antioxidant properties and could effectively inhibit the growth of pathogenic bacteria.

## 4. Functional Food Packaging Based on Nanofibers

Functional food packaging mainly includes antibacterial packaging, antioxidant packaging, barrier packaging, and smart packaging.

### 4.1. Antibacterial Packaging 

The corruption of food is mainly caused by the growth of microorganisms, such as *Listeria monocytogenes* (*L. monocytogenes*), *S. aureus*, and *E. coli*, etc. [129]. Common antibacterial mechanisms include the destruction of cell walls and membranes, the inhibition of efflux pumps known to be responsible for antibiotic resistance, disturbances in the ATP balance that alters energy-mediated cell activities, alterations in protein synthesis and quorum sensing, and pH disturbances, etc., which can all lead to the leakage of intracellular substances and bacterial dissolution [130]. Different bacteria have different resistance to antibacterial compounds due to different cell wall structures. The cell wall is formed by multiple peptidoglycan layers in *S. aureus*, which can prevent the penetration of hydrophobic substances such as essential oils. *E. coli* has a more complex cell wall and is more resistant to hydrophilic compounds. Therefore, essential-oil-based NFMs have greater antibacterial activity against *E. coli* than *S. aureus* [131]. CS is the most commonly used antibacterial material in natural biopolymers. The positive charges of the CS molecule interact with the negative charges of microorganisms, altering the permeability of cell wall and causing leakage of proteins or some important components within the cell, thereby creating an antibacterial effect. In addition, CS with low molecular weight can also enter the cells of microorganisms, inhibiting the growth of cells by restraining the transformation of DNA into RNA [132]. In summary, adding various organic-active drugs (organic antibacterial agents, such as eugenol, CEO, and citric acid) to NFM-based packaging can effectively improve its antibacterial activity. 

Generally, the optimal pH value for bacterial growth is 6 to 7. If it is lower than this value, the growth rate of bacteria will reduce or there will be no growth, and even death. Above this value, the metabolic activity of bacteria will be inhibited and their growth rate will slow down. PLA is a commonly used antibacterial agent, and small molecules of lactic acid are often present in PLA-based NFMs, which can alter the pH value of bacterial media and affect cell growth. Therefore, various active compounds are often incorporated into PLA-based NFMs used in food packaging to ensure that the food has a longer shelf life [133]. Mohammadi et al. [134] electrospun zein/PLA/HPMC/zenian essential oil (ZO) NFMs, and with the increase in ZO content in the NFMs, the NFMs increasingly inhibited the growth of *E. coli* and *S. aureus*. 

Inorganic antibacterial materials are mainly metals or metal oxides, such as CuO, ZnO, iron oxide, and Ag, etc. When they are incorporated into packaging materials, the metal ions diffuse through the packaging, enter the food, and damage the bacterial biofilm, generating ROS and blocking microbial respiration, thus having an antibacterial effect [135]. Ag has high antibacterial activity against a variety of foodborne bacteria. When it acts on food, the growth of fungi and microorganisms will be greatly weakened [77]. Ag NPs can generate ROS, which can bind to cell membranes of bacteria and accumulate in them, leading to cell membrane damage and increasing cell membrane permeability [21]. Jennifer et al. [136] prepared NFMs loaded with Ag and Cu using sepiolite and mesoporous silica as carriers. When spores contact directly with Cu and Ag NPs or composite NFMs, Cu and Ag will damage the growth of the fungus.

### 4.2. Antioxidant Packaging 

When certain substances in food undergo oxidation, the quality of the food will change. Oxidation of proteins reduces the moisture content of meat, and oxidation of lipids causes deterioration of food, leading to sensory changes and nutrient losses in food [137]. Antioxidants can effectively eliminate free radical oxidation at low concentrations, which can directly act on free radicals or indirectly eliminate substances prone to free radicals. Compounds containing polyphenols and phenolic derivatives or organic sulfides are the most commonly used antioxidants, which can scavenge free radicals during the antioxidant process, preventing most organics and polymers from photothermal degradation [138]. Antioxidants generally interrupt the propagation of free radicals in the antioxidant process by scavenging peroxidized components, chelating metal ions, extracting oxygen, and stimulating antioxidant enzyme activity, etc. [139]. Bruni et al. [140] prepared zein/sage-extract NFMs using ES. The sage extract contained a variety of phenolic compounds, which enhanced the antioxidant activity of the NFMs. In antioxidant applications in food packaging, emulsion ES or coaxial ES can better encapsulate active drugs to achieve te slow release of drugs. Bruni et al. [96] electrospun emulsions containing carotenoids, and the obtained NFMs could slow down the release rate of carotenoids after annealing treatment, allowing the antioxidants to act on the food for a long time. In addition to active agents, some polymers also have free-radical-scavenging and antioxidant properties. Bharathi et al. [141] did not use any active agents, and zein was sprayed onto CS NFMs by ES. After contact with food, the zein-based NFMs showed great scavenging activity against free radicals.

### 4.3. Barrier Packaging 

The main purpose of using packaging materials is to set up a barrier between the environment and food. The presence of water, UV light, and oxygen will cause the growth of food microorganisms and lead to food corruption. The porous structure of electrospun NFMs can load active compounds, while active agents absorb water vapor and oxygen generated during food storage, thus improving the barrier properties of packaging [142]. When the water contact angle (WCA) of NFMs reaches over 150°, a superhydrophobic surface is formed, which has the function of self-cleaning. In ES, the hydrophobicity of NFMs is often achieved by enhancing the surface roughness and surface modification [143]. Heat treatment can be used to modify the structure of proteins and protein-based materials, improving the stability of proteins by decreasing the crystallinity and surface-absorbed hydroxyl groups, making the materials insoluble in water [144]. Li et al. [145] used heat treatment to improve the water resistance of CS-based NFMs and found that the WCA of NFMs increased as the heat treatment temperature increased. When the temperature reached 150 °C, the WCA reached 122°, indicating good hydrophobicity. Limonene permeance analysis is often used as a standard for aroma barriers and as an indicator for oxygen barrier properties. Busra et al. [146] used electrospun pectin-based NFMs as the interlayer between two PHBV layers and annealed the whole structure to produce a fully bio-based and biodegradable multilayer film (PHBV/electrospun pectin/PHBV) with enhanced barrier performance against water vapor and limonene. PHBV/electrospun pectin/PHBV exhibited lower permeance values for water vapor (1.75 ± 0.14 × 10^−10^ kg·m^−2^·Pa^−1^·s^−1^) and limonene (0.22 ± 0.11 × 10^−10^ kg·m^−2^·Pa^−1^·s^−1^) than PHBV/PHBV (5.00 ± 0.83 × 10^−10^ kg·m^−2^·Pa^−1^·s^−1^ and 3.81 ± 0.47 × 10^−10^ kg·m^−2^·Pa^−1^·s^−1^). Fabra et al. [147] used electrospun zein NFMs as a high barrier interlayer, which significantly improved the oxygen and water barrier properties of multilayer films. The oxygen permeability of multilayer films prepared by casting and compression-molding methods are effectively improved by the longest deposition time by up to 58% and 76% in films, respectively. Furthermore, the hydrophobicity of gelatin is poor, and the combination of CS and gelatin will form hydrogen bonds between the polymers, reducing the hydrophilic groups in the gelatin, thus improving the hydrophobic properties of NFMs [148,149]. 

In addition to insulation from oxygen, water vapor and heat, NFMs can also provide insulation from UV light by combining them with other materials to create a multilayer structure. Packaging opacity is an important parameter in food applications, and the UV–vis light barrier ability of food packaging is reflected by its opacity. Although PLA has excellent performance, it has high transparency and cannot effectively reduce the adverse effects of UV–vis light on food. Ayca et al. [54] electrospun SPI/HPMC nanofibers onto PLA NFMs, and the double-layer NFMs had a semi-crystalline structure, resulting in a higher opacity of the NFMs. It was suggested to use the double-layer NFMs in packaging of light-sensitive foods. 

### 4.4. Smart Packaging

Smart food packaging is generally used to sense and detect changes in food during transportation and storage, and to report information about the food to the consumer or food manufacturer [150]. It often uses intelligent devices such as data carriers (barcodes and radio frequency identification tags (RFIDs)), indicators (provide food safety and quality information in real time), and sensors (quickly measure food condition) [151]. Leakage indicators (LIs), time–temperature indicators (TTIs), freshness indicators (FIs), and RFIDs are common applications in smart food packaging [152]. In a smart packaging system, materials that interact with the surrounding environment are added to the packaging. When the food quality changes with time, temperature, and freshness, the additional materials will interact with the surrounding substances. The smart packaging can record changes in the external environment and the food during storage, communicating the food’s status to consumers, achieving real-time food condition monitoring [153].

#### 4.4.1. Leak Indicators (LIs)

In order to investigate the changes in sugar, protein, gas, and pH values around food, various sensors are used commercially to detect substance leaks in food. Liu et al. [154] fixed glucose oxidase and porous tin dioxide nanofibers on a Prussian-blue-modified gold electrode. When the glucose was oxidized, the electrode could quickly capture its chemical signals and convert them into electrical signals, thus realizing the detection of the glucose concentration. However, commercial sensors are costly and specialized in operation, meaning they are not easy for consumers to observe. In order to facilitate consumers to intuitively monitor the leakage of various substances in food, some studies have prepared other simple and intuitive smart packaging. 

**Figure 5:**
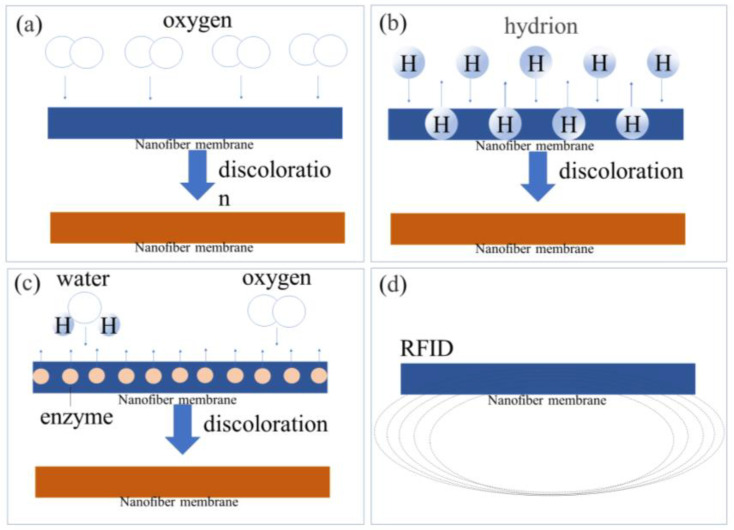
Smart packaging mechanisms: (**a**) redox reactions in LIs; (**b**) protonation and deprotonation in FIs; (**c**) enzymatic catalysis in TTIs; (**d**) electromagnetic fields of RFIDs.

Redox reactions are a common chemical reaction which can change the color of the indicator in smart packaging to express variations in food freshness during storage (Figure 5a). The shelf life of food can be extended when the concentration of gas in the package is kept in balance. However, during the aerobic and anaerobic respiration of food, the gas balance is broken, allowing the gas indicator to directly observe the changes in gas in the environment during the deterioration of food [155]. In smart packaging, oxygen indicators have been developed to monitor the concentration of oxygen in the food environment. The combination of the indicator with oxygen results in a redox reaction and produces a new compound that detects the amount of oxygen in the package. Yilmaz et al. [156] prepared an oxygen indicator using a chemical reaction between TiO_2_ and methylene blue (MB). The electrospun PVA/MB/TiO_2_ NFM was coated with PS and used to prevent leakage of dye from the indicator. In the experiment, it was found that when the oxygen content in the food environment changed, there would also be significant color changes in the packaging.

#### 4.4.2. Freshness Indicators (FIs)

Generally, when food is kept fresh, it has a stable pH range. During food storage, the influence of the surrounding environment, decomposition of internal substances, cellular respiration, as well as the release of organic acids, carbon dioxide, glucose, and other compounds in food will lead to changes in the food’s pH values. FIs consist of polymer materials and pH-sensing colorants which will change color when the food quality changes by different degrees [157]. In acidic and alkaline environments, some active compounds are prone to protonation and deprotonation, leading to structural changes in conjugated double bonds and lone electron pairs, thus causing color changes in FIs (Figure 5b). Natural active compounds such as anthocyanins and curcumin can be used to create FIs, which provide consumers with information about food freshness by changing the color of indicators on the packaging when the food is in direct contact with FIs [106]. 

Anthocyanins exhibit different molecular structures at different pH values. When the pH is below 2, flavylium cations are the main structure, showing red; when the pH increases, anthocyanins undergo deprotonation, and the double bond of flavylium cations is extended conjugated in the surrounding environment, forming colorless carbinol pseudobase and chalone, thus appearing pink; with an increase in the pH, flavylium cations are further deprotonated to form purple quinones, making anthocyanins purple and blue [157]. Liu et al. [106] used electrospun PCL/anthocyanin NFMs as a pH indicator to monitor the freshness of shrimp. When the pH value was 2, the color of NFMs showed red, and when the pH value increased from 3 to 10, the color of NFMs changed from blue to green. The pH value of fresh shrimp was 7.1. Over time, the pH value of the shrimp increased, and the color of the NFMs gradually changed from blue to light green. Furthermore, the pH value of some foods decreases as they deteriorate, however, the color change in anthocyanins from neutral to weakly acidic environments is not obvious. Gao et al. [158] added Fe ions to electrospun NFMs with anthocyanins in order to detect the freshness of acidic spoiled foods such as milk, and they combined with each other to form a colored chelate. The chelate showed more pronounced discoloration than the pure anthocyanin indicator. When the pH changed from 7 to 3, the color of the indicator changed significantly from purple-black to blue-violet and light purple-red.

Under alkaline conditions, curcumin can ionize phenol oxygen ions. With the increase in pH, the hydroxyl groups at both ends of curcumin will have a conjugated effect of electron cloud deviation, forming a conjugated system. Generally, the larger the conjugated system, the higher the extinction coefficient, the stronger the absorption, and the darker the color expression. Therefore, the color of curcumin gradually deepened with the increasing pH. Yildiz et al. [159] prepared curcumin/CS/PEO NFMs using ES, which showed different colors at different pH values and could monitor chicken freshness. When the chicken packaged with the NFMs deteriorated, the pH value increased and the color of the NFMs changed significantly, from yellow to orange. 

#### 4.4.3. Time–Temperature Indicators (TTIs)

TTIs respond to temperature changes in the environment [160], and can show changes in the temperature and quality of food throughout the entire supply chain from production to storage and distribution. TTIs are mainly achieved through the use of polymerization, photochromism, indicators, and enzyme catalysis [161]. The principle of an enzyme TTI is to hydrolyze the substrate to cause pH changes, or to catalyze redox and other reactions to form colored products, causing color changes in the TTI (Figure 5c) [162]. Laccase is the most common redox enzyme. Under the effect of enzyme catalysis, the redox reaction causes chemical reactions of phenolic compounds in the substrate, such as aminophenols, polyphenols, and phenols, forming new substances that cause a color change in the indicator. Jhuang et al. [163] immobilized laccase on an electrospun zein NFM. This composite NFM for TTIs had ideal tolerance and temperature sensitivity, requiring 26 days to reach the coloring end point at 4 °C. TTIs can also be applied in various processes of food transmission in the cold chain, enabling managers to monitor food quality more intuitively and clearly. Although the enzyme TTI system has a high response and accuracy to temperature changes, it is unstable and inefficient in practical applications. Based on this problem, Lin et al. [164] fixed laccase on electrospun CS NFMs to improve its stability. In addition, dispersive substances and enzyme inhibitors were added to reduce the pigmentation caused by long-term enzyme coloring. 

#### 4.4.4. Smart Tags

Carbon-containing materials are widely used in the field of electronics due to their good mechanical properties, as well as excellent electrical and thermal conductivity. When combined with other high-performance polymers, carbon-containing materials can be applied to manufacture smart tags on packaging, such as barcodes and RFIDs [165]. RFIDs use electromagnetic fields to store and transmit real-time information about products, achieving automatic identification and traceability of products. The tags consist of an integrated circuit built into an antenna, which can transmit information stored in the chip to a reader, thereby simultaneously monitoring multiple items and storing diverse information [150] (Figure 5d). Poly (ethylene-co-vinyl alcohol) is often used as the inner layer of high barrier packaging, which can also respond uniquely to electrical stimuli, providing relevant information about the physicochemical properties of the packaged food. Torres et al. [116] embedded graphene nanosheets into a poly(ethylene-co-vinyl alcohol) NFM, and the composite NFM displayed conductivity, meaning it could be applied in smart tags with high electrical rate capabilities.

## 5. Application of Functional Packaging Based on Nanofibers in Different Foods

**Figure 6:**
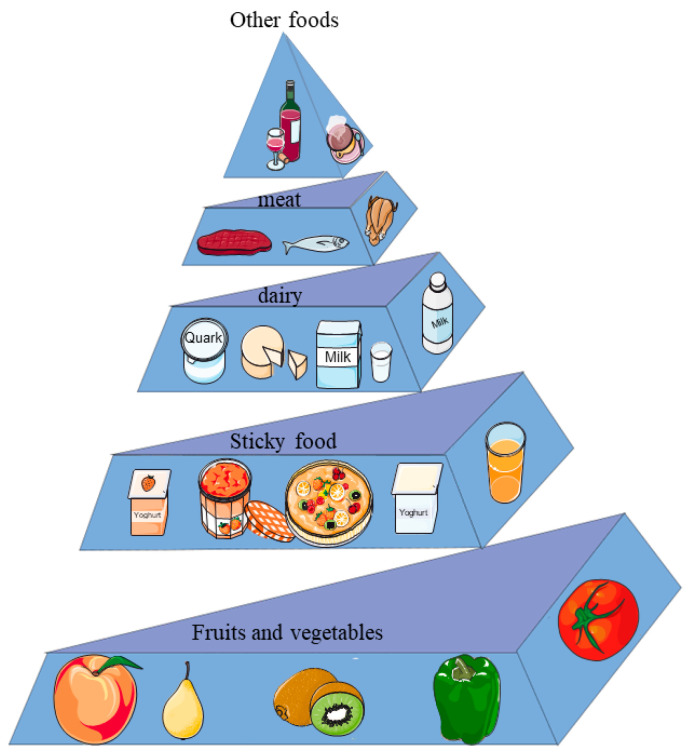
Different foods.

Different foods (Figure 6) may be corrupted and deteriorate for different reasons. Fruits and vegetables are prone to release ethylene during storage, which accelerates their ripening and eventual decay. Meat is prone to oxidation during storage, leading to changes in color and flavor. Sticky foods such as honey can easily stick to the packaging during storage, causing food loss. Temperature-sensitive foods such as sausages and other cooked foods are more sensitive to external temperature during storage, and it is easy for them to deteriorate when the temperature is too high. During the storage of food, the growth of microorganisms will lead to the deterioration of food. Different functional food packaging based on nanofibers has been designed for different foods to extend their shelf life and monitor their quality. 

### 5.1. Fruits and Vegetables

**Figure 7:**
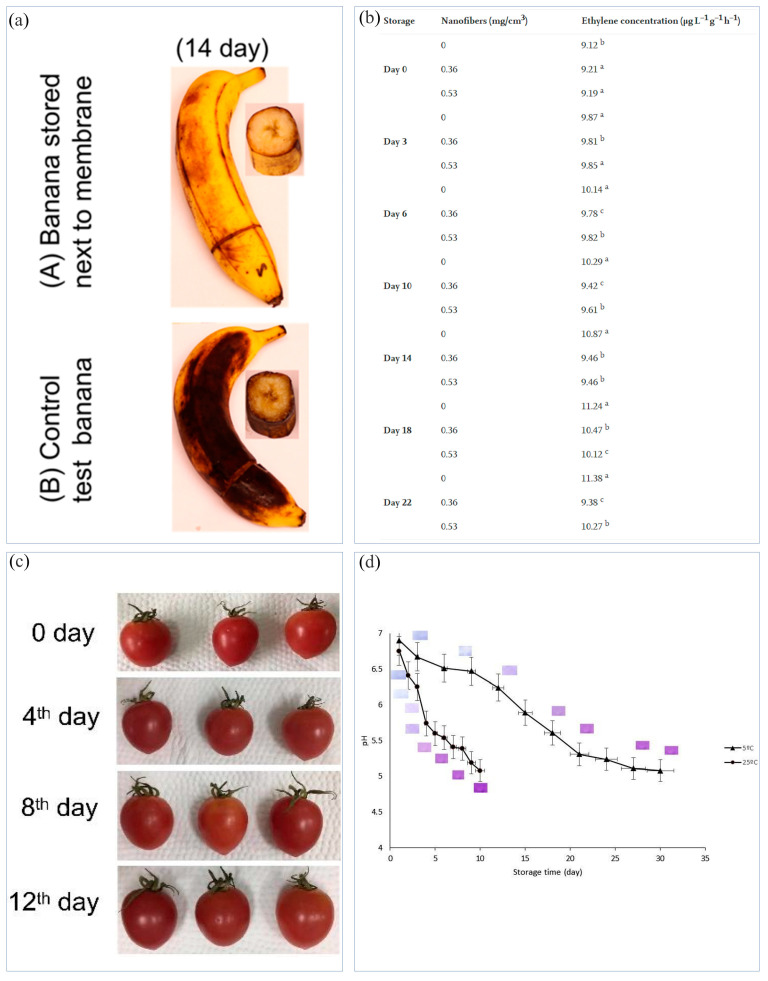
(**a**) Quality of bananas after placing different films next to them for more than ten days [168]; (**b**) photocatalytic activity of nanofibers with 5% TiO_2_ against ethylene during storage of cherry tomato fruits for 22 days [170]; (**c**) package application of cherry tomatoes: PEO-RE (core)/zein-AgNPs-RE (sheath) coaxial nanofiber packaged group [120]; (**d**) pH changes in the Rutab fruit and their relationships with the color changes in the pH indicator with storage time [171].

Ethylene is a ripening agent that can accelerate plant maturation and senescence. Ethylene in fruits can accelerate the degradation rate of chlorophyll, leading to fruit softening and shortening its shelf life. When using some special drugs to inhibit ethylene, irreversible decomposition products are easily generated, which may cause toxicity [166]. When adsorbents such as activated carbons act on ethylene, their adsorption effects are limited and will not cause chemical damage to ethylene [167]. Potassium permanganate is a commonly used strong oxidant in the fruit industry which can oxidize and degrade ethylene. Tirgar et al. [168] prepared PAN NFMs containing alumina NPs using ES, and cast potassium permanganate on the NFMs. The prepared NFMs were placed beside bananas. After more than 10 days, compared with the control group, it was found that the bananas placed beside the NFMs containing potassium permanganate were fresher (Figure 7a). However, potassium permanganate will be reduced and deactivated after contact with common polymers and solvents during the ES process. In addition, potassium permanganate may pollute the environment [169]. Therefore, based on safety and environmental protection, the use of photocatalysis to degrade ethylene released from food is often studied. When the active packaging is exposed to UV or visible light, the photocatalyst in the packaging can effectively degrade ethylene into water and carbon dioxide [135]. TiO_2_ is one of the best photocatalysts, with the advantages of chemical inertness, being environmentally friendly, and low cost. Under the irradiation of UV light, it can generate a lot of hydroxyl radicals, which can effectively degrade ethylene. Zhang et al. [169] prepared TiO_2_/PAN NFMs and found that with the increase in TiO_2_ content, the decomposition rate of ethylene increased. Bruna et al. [170] electrospun zein/TiO_2_ NFMs as packaging to store cherry tomatoes. Compared to the control group, the tomatoes in the packaging containing TiO_2_ showed lower ethylene concentrations, demonstrating that the NFMs had photocatalytic activity against ethylene for 22 days during storage (Figure 7b). 

The destruction of fruits and vegetables by bacteria can lead to the fermentation of sugars in them to form lactic acid, aldehydes, and carbon dioxide, etc., resulting in food corruption and odor, as well as changes in the pH value [172]. Therefore, it is necessary to use antibacterial packaging to resist microbial erosion, thereby slowing down the decline in food quality and extending the shelf life of food. Jiang et al. [120] prepared core-shell nanofibers using coaxial ES using resveratrol as the core as well as Ag NPs, zein and resveratrol as the shell. When wrapping cherry tomatoes with the NFMs, it was found that the fruit surface could remain flat and smooth for 12 days without mold (Figure 7c), while the cherry tomatoes in the control group still grew mold due to the absence of antibacterial packaging. Wen et al. [173] prepared PVA/CEO/β-cyclodextrin NFMs to wrap strawberries, which showed good antibacterial activity against *S. aureus* and *E. coli*. The strawberries packed with PVA/CEO/β-CD NFM showed no sign of decay even at day 6. Motahira et al. [73,75] electrospun hydroxycellulose/PVA/polyvinyl pyrrolidone NFMs, which were used to cover vegetables for 10 days and fruits for 40 days without significant damage to the morphology and quality of the food. Min et al. [127] used pectin-loaded thymol to embed into PLA fibers with a porous structure, and the pectinase secreted by microorganisms could degrade pectin as a stimulating factor to achieve accurate control of the continuous and effective release of thymol. The prepared NFMs could effectively inhibit the mold on citrus and prolong its shelf life. In addition, FIs can be used to monitor the freshness of fruits and vegetables. Maftoonazad et al. [171] electrospun NFMs based on PVA and red cabbage extract. With the decrease in the pH value, the color of the NFMs changed from light purple to dark purple, which was used to detect the freshness of jujube (Figure 7d). 

### 5.2. Meat Packaging 

**Figure 8:**
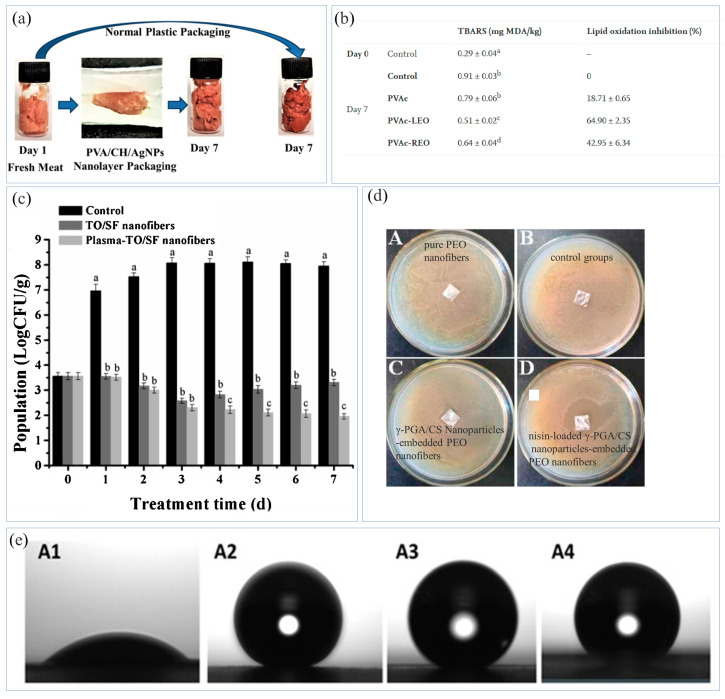
(**a**) Application of polyvinyl alcohol/chitosan/Ag nanoparticles composite nano-layer for packaging of fresh meat [122]; (**b**) thiobarbituric acid reactive substances (TBARSs) and estimated lipid oxidation inhibition in fresh chicken breast fillets (day 0) and in the packaged samples after 7 days of storage [128]; (**c**) antibacterial activity nanofibers in agar-diffusion assay [73]; (**d**) the inhibition zones of nanofiber membranes [176]; (**e**) shape of the water droplets on (**A1**) paper, (**A2**) paper/polylactic acid/SiO_2_, (**A3**) paper/polycaprolactone/SiO_2_, (**A4**) paper/poly (3-hydroxybutyrate-co-3-hydroxyvalerate)/SiO_2_ [177].

Meat contains high levels of protein, which can supply nutrition for the human body. However, meat is prone to corrosion and oxidation of lipids and proteins during storage, resulting in sensory changes in the meat. In addition, meat is susceptible to microbial infections, and the growth of harmful microorganisms will lead to food corruption, even threatening the lives of consumers in serious cases [174]. High-fat meat is more sensitive to lipid oxidation, and the high concentration of oxygen will keep the meat bright red. The oxidation of proteins may reduce the quality of fresh meat [175]. In an experiment, meat was crushed in a grinder, and after seven days the samples placed in the composite NFMs showed better sensory qualities than the control group samples [122] (Figure 8a). This meant that when packaging meat, the NFMs partially isolated the meat from oxygen to inhibit the growth of bacteria and ensure the quality of the food. 

Furthermore, bacteria breed easily in some meat products during their processing and distribution, leading to food deterioration and waste. *L. monocytogenes* is a food borne Gram-positive pathogen that usually grows in meat. Vivek et al. [122] prepared composite NFMs by blending CS with Ag NPs and PVA through ES. It was found that compared to traditional plastic packaging, the NFM packaging showed better antibacterial activity, with fewer bacteria growing in the samples. Goksen et al. [128] prepared PVA/citric acid/essential oils NFMs, and found that the NFMs had antibacterial effects against *L. monocytogenes*. Chicken breasts packaged with the NFMs could inhibit lipid oxidation (Figure 8b). Therefore, the degree of discoloration of the chicken breasts packaged with the NFMs was small, effectively ensuring its appearance quality during storage and extending its shelf life. *Salmonella* is also generally considered as the most important pathogen causing foodborne epidemics. It is related to the pollution of meat, especially poultry, and can grow in some extreme environments. Lin et al. [73] prepared thyme essential oil/SF NFMs using ES, and conducted plasma treatment on the NFMs. After packaging chicken with the NFMs, the number of *Salmonella typhi* declined dramatically (Figure 8c). *Pseudomonas fluorescens* (*P. fluorescens)* and *Vibrio parahaemolyticus* (*V. parahaemolyticus*) exist widely in aquatic products and tend to cause spoilage and deterioration of food. Shi et al. [178] used ES to encapsulate the complex of octyl gallate and cyclodextrin (OG/βCD) into PLA, and found that this encapsulation was effective in disrupting the cell wall, penetrating into the cell and generating hydroxyl radicals, thereby exerting antibacterial effects and effectively inhibiting the growth of *P. fluorescens* and *V. parahaemolyticus*. The shelf life of fish fillets could be extended to 15 days at 4 °C by wrapping them with the NFMs. Compared with the sample without NFM packaging, the packaged fish fillets were fresher. Mutton is easily decomposed by microorganisms during storage, producing biogenic amine, which will react with nitrite to produce nitrous acid, causing cancer. Therefore, some studies have designed detection methods for biogenic amine [179]. Sun et al. [180] prepared anthocyanin/PLA NFMs, which initially appeared pink. With the increase in amine content, the color of the NFMs changed from pink to colorless, accurately detecting the deterioration of mutton.

### 5.3. Dairy Products

Cheese is a common dairy product that is widely appreciated by consumers around the world. However, it is highly susceptible to contamination by various pathogens during processing and storage, such as *L. monocytogenes*, *S. aureus*, *E. coli*, and so on. In the production process of dairy products, natural preservatives such as bacteriocins are often used to control the growth of fungi. Nisin is a bacteriocin with excellent antibacterial activity. Cui et al. [176] studied and designed PEO NFMs containing nisin-loaded poly-gamma-glutamic acid/CS (NGC) NPs. Compared to pure PEO NFMs, the PEO NFMs embedded with NGC NPs showed more significant antibacterial activity against *L. monocytogenes* (Figure 8d), and did not change the sensory quality of the cheese products. Goksen et al. [181] prepared zein/laurel essential oil/rosemary essential oil NFMs using ES. The effects of zein NFMs loaded with two essential oils on reducing microbial growth on the cheese surface were evaluated, and it was found that the effective release of the two oils was prolonged after encapsulation by nanofibers, which effectively inhibited the growth of *L. monocytogenes*, *S. aureus*, and total mesophilic bacteria on the cheese slices. In addition, enzyme TTIs can be used for real-time monitoring of quality changes in dairy products during storage. Tsai et al. [182] fixed laccase on CS/PVA/tetraethyl orthosilicate NFMs, and used guaiacol as a colorant to detect the quality of milk. The color of the NFMs changed from light brown to dark brown, and finally, to purplish brown, which was used for real-time monitoring of changes in milk quality during storage.

### 5.4. Sticky Food

Sticky foods such as honey, jam, and yogurt often adhere to the packaging during the packaging process, resulting in a loss in food quantity. In the food industry, glass and metal products are generally used to preserve such foods, but such packaging is usually non-degradable. In order to achieve the purposes of self-cleaning, waterproof, and moisture-proof packaging and extending the food shelf life in packaging, the preparation and development of hydrophobic NFMs has gradually increased [183]. Currently, plasma treatment, grafting, and ES methods are commonly used to modify NFMs or form multilayer structured NFMs to make them obtain hydrophobicity [184]. Vinod et al. [185] prepared gum arabic (GA) NFMs, and conducted γ-ray irradiation and metal plasma on them to improve the hydrophobicity of the NFMs. Because of its self-cleaning, water repellent, antibacterial, and moisture-proof properties, superhydrophobic cellulose paper has a variety of potential food application prospects, meaning it can be widely used in microwave food, fast food, and beverage packaging. Lafraya et al. [177] sprayed PLA, PCL, and PHBV solutions onto cellulose paper using ES, and then electrosprayed silica particles on the polymer layer to prepare three multilayer superhydrophobic bio-papers. The synthesized bio-papers all showed an increase in WCA and a decrease in water vapor permeability after annealing, with the WCA reaching a maximum of over 150° (Figure 8e), realizing the manufacture of superhydrophobic bio-papers. When packaging sticky food, the superhydrophobic bio-papers showed a high level of super-repulsion to the sticky food.

### 5.5. Others

Some foods, such as cooked food, ice cream, and cold drinks, cannot be kept at high temperatures for a long time. It is necessary to maintain a constant temperature during storage and transportation to prevent food from spoiling due to changes in external temperature. Phase change materials (PCMs) can absorb or release energy under melting and crystallization conditions to buffer temperature changes in the external environment, hence they are often used to manufacture energy storage NFMs. Amparo et al. [118] encapsulated Rubitherm-RT5^®^ (RT5) in PCL, PLA, and PS NFMs. The PCL NFMs containing RT5 could maintain a temperature of 4–6 °C within 1.5 min, and showed a certain temperature buffering ability when the external temperature changed, which could improve the safety of food storage. Temperature variations affect the growth of microorganisms, and some sausages with a high nutrient content are susceptible to temperature fluctuations during storage and transport. Therefore, controlling temperature variations has a significant impact on the quality and flavor of sausages. Karimc et al. [59] used tedecane as a PCM and cinnamaldehyde as an antibacterial agent to form temperature-buffering and antibacterial NFMs. Under the same conditions, when NFMs loaded with PCM were used to package sausages, it took 8 h for the sausages’ core temperature to rise from 2 °C to 7 °C, while the core temperature of sausages packaged with commercial polyamide reached 7 °C within just 2 h. In addition, the peroxide, thiobarbituric acid (TBA) value, and the number of *E. coli* in the sausages packaged with NFMs containing tetradecane were lower than those in the control group. This indicated that during food storage, the NFMs loaded with PCMs not only inhibited the growth of microorganisms and the occurrence of chemical reactions, but also reduced the need for the use of antibacterial agents.

## 6. Conclusions

Electrospinning (ES), as a commonly used simple and efficient method for preparing functional nanofibers, can control the morphology, structure, property, and function of nanofibers by modifying devices, adjusting process parameters, and loading various functional substances into nanofibers. This makes electrospun NFMs with large specific surface area and high porosity show great application potential in the field of food packaging. Many reliable, safe, and biodegradable raw materials in nature can be used for food packaging production using ES technology, and various fillers can be loaded into the base materials to enhance the mechanical, optical, pH sensitive, antimicrobial, and antioxidant properties of food packaging, thus obtaining food packaging with different functions. Meanwhile, the structural design of the nanofibers can also endow food packaging with different functions. In order to obtain antibacterial and antioxidant packaging, various active drugs or NPs (such as antibiotics and antioxidants) are encapsulated in nanofibers through ES, effectively extending the shelf life of food. Moreover, in order to monitor the quality of food in real time, various smart packaging materials use indicators (such as LIs, TTIs, FIs, and RFIDs) to react with oxygen, carbon dioxide, glucose, and other substances released from food, causing the discoloration of packaging to alert monitors of food quality problems. Simultaneously, the application of functional packaging based on electrospun nanofibers in different foods is summarized. 

However, there are limitations to the commercial application of electrospun nanofibers in food packaging. At present, the application of electrospun nanofibers in food packaging is still mainly concentrated in the laboratory due to its low productivity. In addition, although natural biopolymers are green, safe, and non-toxic, they are hydrophilic and their barrier properties are weakened at high humidity, which limits their application in food packaging. Moreover, the small-sized nanofibers may cause migration of their components into food products during packaging, which may lead to environmental contamination and even affect human health. Therefore, a systematic risk assessment is necessary. Currently, various functional nanofibers have been widely studied in food packaging, but based on the aforementioned shortcomings, electrospun nanofiber-based packaging still needs further development in the future.

## Figures and Tables

**Table 1:** ES techniques and their yields.

ES Method	Fiber Material	Spinning Voltage	Yield	Fiber Diameter
Single-needle ES [29,40]	Polyvinylpyrrolidone (PVP)	15–60 kV	0.01–0.1 g/h	75–1500 nm
Multi-needle ES [41]	PVP	15 kV	Several times of SNES	50–100 nm
Coil ES [42]	Polyacrylonitrile (PAN)	60 kV	10–23 g/h	200–400 nm
Bubble ES (BE) [43]	Polyvinyl alcohol (PVA)	35 kV	3 g/h	–
Bowl ES [44]	Poly (ethylene oxide) (PEO)	16 kV	0.684 g/h	243–293 nm
Free surface ES [45]	PAN	70 kV	100 times of SNES	488–576 nm
Tipless ES [46]	PEO	50–80 kV	260 times of SNES	207–453 nm
Needleless vertical rod ES [47]	PVA	30–50 kV	0.36–1.92 g/h	357–383 nm
ES with a helically probed cylinder [48]	PAN	15–17 kV	3.2 g/h	2–800 µm
Modified BE [49]	PVA	30–70 kV	19.8–72 g/h	108–168 nm
Modified BE [50]	Silk fibroin (SF)	50 kV	3.1 g/h	50–313 nm

**Table 2:** Electrospun nanofiber materials and properties for food packaging.

Natural Polymers	Synthetic Polymers	Various Fillers	Application Effect	Reference
Zein		Tetradecane, cinnamic aldehyde	Good antibacterial effect and long retention time at low temperature	[59]
Zein		Chilto extracts	Antioxidant	[53]
Zein	Polyoxyethylene	Ag NPs, resveratrol	Good antibacterial effect (the diameter of inhibition zone: 7.26 ± 0.10 mm against *E. coli*, 8.89 ± 0.09 mm against *S. Aureus*)	[60]
Zein		Curcumin	Good antibacterial and antioxidant properties	[61]
Zein, alginate		Basil oil (Ocimum basilicum)	Good antibacterial effect and excellent mechanical properties	[62]
CS	PVA	Ag NPs	It is hydrophobic (WAC: 95.82 ± 10.27°) and can effectively resist bacteria (the diameter of inhibition zone: 20.0 ± 0.7 mm against *E. coli*, 21.0 ± 0.6 mm against *L. Monocytogenes*)	[63]
CS	Polycaprolactone	Rutin	The fiber becomes thinner and the antibacterial property becomes better	[64]
CS	PVA		Increase in thermal stability and mechanical properties, and decrease in water permeability (WAC: 109.45 ± 3.88°)	[65]
CS	PEO	Microalgal phenolic compounds	Good antibacterial property	[66]
CS, gelatin	3-phenyllactic acid		It has good thermal stability and water vapor permeability, and can effectively resist bacteria	[67]
Gelatin		Angelica essential oil (AEO)	It improves the hydrophobicity (WCA: 101.3 ± 5.55°) and shows excellent antioxidation (radical scavenging rate: 85% ± 6%) and antibacterial properties	[68]
Gelatin		Lemongrass essential oil	Good antibacterial effect	[69]
SPI, hydroxypropyl methylcellulose	PLA		Reduced transparency and good thermal stability (water vapor permeability: 6.225 ± 0.313 × 10^−11^ gm^−1^ s^−1^ Pa^−1^)	[54]
SPI, ethyl cellulose		Bitter orange peel extract	Antibacterial and antioxidant	[70]
Starch		Tea polyphenols	Good oxidation resistance, hydrophobic	[71]
Starch		Thyme essential oil (TEO)	Good antioxidant activity	[72]
SF	PEO	TEO	Uniform fiber morphology	[73]
CNC	PLA	Lauryl arginine ethyl ester (LAE)	Good antibacterial effect	[74]
CMC	PVA, PVP		Good air permeability	[75]
Pectin	PLA	Thymol	Good antibacterial effect (95% inhibition rate against *E. coli*, *S. aureus* and *Bacillus subtilis*)	[76]
CA	PVC	Ag NPs	Good stretching effect and excellent antibacterial effect	[77]
Beta-cyclodextrin		Cinnamon essential oil (CEO)	Good antibacterial effect and hydrophobic (WCAs of all films were above 90°)	[78]
Hydroxypropyl-beta-cyclodextrin		Cuminaldehyde	Good antibacterial property	[79]
Regenerated cellulose		Carboxylated carbon nanotubes, graphene oxide	Good antibacterial effect	[80]
HPMC	PEO	Gallic acid	Improved antibacterial and antioxidant properties	[81]
	PVA	Ag NPs	Good antibacterial property (the diameter of inhibition zone: 21.47 ± 0.15 mm against *S. aureus*)	[82]
	PVA	Essential oils from two broadly used spices (Laurus nobilis (LEO) and Rosmarinus officinalis (REO))	Good antioxidation and antibacterial properties, good thermal stability, and water resistance	[83]
	PVA	Clove oil (CO), citric acid	Improvement of thermal stability and mechanical properties	[84]
	PCL	Vitamin E	Good oxidation resistance	[85]
	PCL	Ag NPs	Good antibacterial property and hydrophobic (WCA: 134°)	[86]
	PHBA	ZnO NPs	Good antibacterial effect	[87]
	PEO	Aloe vera extract	Good oxidation resistance	[88]

## Data Availability

All data generated or analyzed during this study are included in this published article.
